# The Panhandle Formed by Influenza A and C Virus NS Non-Coding Regions Determines NS Segment Expression

**DOI:** 10.1371/journal.pone.0081550

**Published:** 2013-11-21

**Authors:** Bernadette Crescenzo-Chaigne, Cyril Barbezange, Sylvie van der Werf

**Affiliations:** 1 Unité de Génétique Moléculaire des Virus à ARN, Institut Pasteur, Paris, France; 2 Unité Mixte de Recherche 3569, Centre National de la Recherche Scientifique, Paris, France; 3 Unité de Génétique Moléculaire des Virus à ARN, Université Paris Diderot Sorbonne Paris Cité, Paris, France; University of Hong Kong, Hong Kong

## Abstract

Exchange of the extremities of the NS segment of type A and C influenza viruses in reverse genetics systems was used to assess their putative role in type specificity. Restoration of each specific proximal panhandle was mandatory to allow the rescue of viruses with heterotypic extremities. Moreover, the transcription level of the modified segment seemed to be directly affected by the distal panhandle strength.

## Introduction

Influenza A, B and C viruses are members of the *Orthomyxoviridae* family, a group of enveloped, segmented, single-stranded negative-sense RNA viruses. Reassortment between type A, B and C influenza viruses has never been reported to date. The general structural features and genome organization of influenza viruses suggest that they share a common ancestor [1]. The genome of influenza A and B viruses consists of eight segments, whereas that of influenza C virus has only seven segments since it has a single envelope glycoprotein (HEF) instead of two for type A and B viruses (HA and NA) [2]. 

The genomic viral RNAs (vRNAs) are associated with the nucleoprotein (NP) and the polymerase complex (P). The latter is formed by three subunits named PB1, PB2 and PA for influenza A and B viruses and PB1, PB2 and P3 for influenza C virus, respectively. In the nucleus of infected cells, viral messenger RNA (mRNA) synthesis is initiated with capped RNA primers that are cleaved from host cell mRNAs (cap-snatching mechanism), and terminates 17 to 22 nucleotides (nt) upstream of the genomic vRNA template 5’ end at a stretch of five to seven uridine residues used as a polyadenylation signal. Genomic vRNA replication requires a full-length positive-sense RNA template (complementary RNA or cRNA), and both cRNA and vRNA syntheses are primer-independent [2]. 

The coding region of each genomic vRNA is flanked by non-coding (NC) sequences that are divided into conserved and non conserved parts [3]. The length of the NC sequences differs for each segment and also varies between virus types [4,5]. For the 3’ and 5’ ends, respectively, the conserved parts are 12 and 13 nt for type A, 12 and 11nt for type B and 11 and 12 nt for type C influenza viruses [6–8]. The 5’ and 3’ NC sequences base-pair to form two elements: the proximal element or region I (nt 1-9 of the 3’ and 5’ ends) and the distal element or region II involving sequences downstream of nt 10 and 11 from the 3’ and 5’ ends, respectively [9]. Two main secondary structures have been described: the “panhandle structure” resulting from complete base-pairing between the two ends [10–14] and the “corkscrew structure”, where the proximal element form hairpin loops [15–18]. These conformations are known to be critical for transcription and replication of the vRNAs [9]. 

To investigate whether, or not, and how the complete NC regions of a given segment are involved in type specificity, we attempted to rescue, by reverse genetics, type A and C influenza viruses with chimeric non-coding sequences. All experiments were based on the NS segment, the smallest segment for both influenza virus types and for which the NC regions of both viruses are quite similar in length. We showed that type specificity of the proximal element is critical to rescue infectious viruses, and that the distal element might modulate viral transcription. 

## Materials and Methods

### Plasmids and reverse genetics

The 12- or 11-plasmids based reverse genetic systems were used to produce recombinant type A (A/WSN/33) and type C (C/JHB/1/66) influenza viruses, and were adapted from previously described procedures [19–21]. The modified NS plasmids (chimeric) were constructed by PCR. Point mutations in the NS NC regions were introduced by directed mutagenesis using Quikchange II site-Directed mutagenesis kit (Agilent Technologies) according to the manufacturer’s instructions. The primer sequences will be provided upon request. All plasmids were sequenced using a Big Dye terminator sequencing kit and an automated sequencer (Perkin-Elmer).

### Cells and viruses

Human skin melanoma cells (SK93/2) [22], 293T human embryonic kidney cells and Madin-Darby canine kidney cells (MDCK) cells were cultured in DMEM supplemented with 10% and 5% fetal calf serum (FCS), respectively. All cells were grown at 37 °C with 5 % CO_2_. Plaque assay using an agarose overlay was performed for titration of influenza A viruses in DMEM with 1 µg/ml of L-1-tosylamido-2-phenyl chloromethyl ketone TPCK-trypsin (Worthington) for 3 days at 35 °C. Titration by plaque assay of influenza C viruses on MDCK cells at 33°C was described previously [21].

### Virus cloning, amplification and growth kinetics

For influenza A viruses, the rescued viruses were plaque purified twice on MDCK cells before final amplification on MDCK cells at an m.o.i. of 0.01. Growth kinetics were performed with the stock viruses. MDCK cells were infected at an m.o.i. of 0.001 in DMEM supplemented with 1µg/mL TPCK-trypsin. The rescued influenza C viruses were plaque purified on MDCK cells supplemented with bovine brain gangliosides (BBG) before amplification on SK cells. For growth kinetics, SK cells were infected at an m.o.i. of 0.001 in DMEM supplemented with 0.25 µg/mL TPCK-trypsin. Supernatants were collected at indicated time points and virus titres were determined by plaque assay. 

### Determination of 3’ and 5’ end NC sequences of NS genomic RNA segments

Viral genomic RNA was extracted using QIAamp Viral RNA Mini kit (Qiagen) according to the manufacturer’s recommendations and cDNA, PCR and sequencing were performed as previously described [10]. All primer sequences are available from the authors upon request.

### Single-cycle growth kinetics and detection of intracellular viral RNAs

MDCK cells were infected at an m.o.i. of 2 with the wild-type or mutated influenza A viruses. At six hours post-infection, total RNAs were extracted, reverse transcribed using the 3’ universal primer for vRNA (5’AGGGCTCTTCGGCCAGCRAAAGCAGG) or oligo dT for mRNA, and used as a template for quantitative PCR (qPCR). Separate PCRs were performed with segment-specific primers designed by Marsh et al [23] using of a LightCycler 480 (Roche). The relative concentrations of vRNAs and mRNAs were determined by calculating mRNA/vRNA ratios using the crossing point values (Cp). 

As control, a one step qRT-PCR targeting the GAPDH sequences was performed [24].

### Western blot

Cells were solubilised in LDS sample buffer (Invitrogen) complemented with β mercaptoethanol, and proteins were separated by polyacrylamide gel electrophoresis (NUPAGE Invitrogen) followed by western blotting. Rabbit polyclonal anti-NS antibody was a kind gift from D. Marc (INRA, Tours, France). The rabbit anti-PR8, used to detect NP, was produced in house [25]. Anti beta-actin (Abcam, Cambridge, UK) antibody was used for loading controls. 

## Results and Discussion

**Figure 1:**
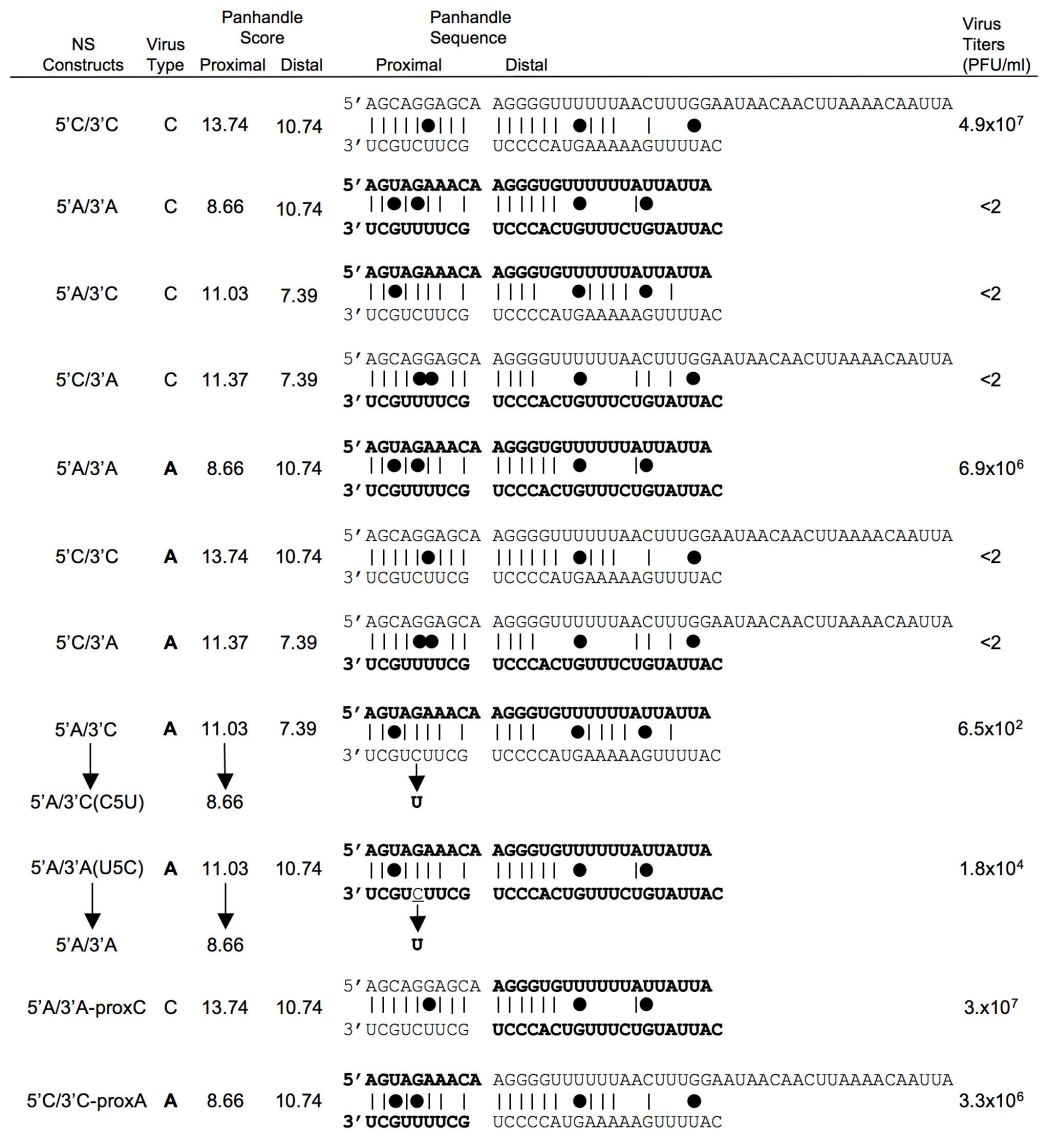
Rescue of influenza viruses harbouring substitutions in the NC region of genomic NS segment. Nucleotide sequences and predicted conformations of the NC region panhandle of the different NS genomic segments tested in type C (in plain, based on C/JHB/1/66) and type A (in bold, based on A/WSN/33) influenza virus reverse genetics systems [20,21]. The introduced mutation in 5’A/3’A(U5C) is underlined. The sequence of both ends of each segment of each rescued virus was verified as described [10], and no mutation was detected except at position 5 in the 3’ end for 5’A/3’C and 5’A/3’A(U5C) yielding viruses 5’A/3’C(C5U) and 5’A/3’A, respectively (indicated by arrows). In constructs 5’A/3’A-proxC in the type C system and 5’C/3’C-proxA in the type A system, only the distal panhandle was modified, but the homotypic proximal panhandle was conserved. The energy barriers of the canonical pairs C:G and U:A, and of the wobble base pair G:U (represented by a black dot) were described by Vendeix et al [34] and were used to calculate a score to evaluate the panhandle strength. The proximal panhandle consists of 9 potential base-pairs, when the distal panhandle was defined to include the potential base-pairs up to the type A poly-U signal. Type C reverse genetics was performed in 293T cells and supernatants were collected and titrated at day 10 post-transfection (p.t.). Type A reverse genetics was performed in a co-culture of 293T and MDCK cells, and supernatants were collected and titrated at 72h p.t. Titers in PFU/ml are the mean of 2 to 4 independent experiments.

The role of NC sequences in type specificity was investigated for the NS segment of the most divergent type A and C influenza viruses using reverse genetics systems [19–21]. A set of type A and type C chimeric viruses was generated, in which one or both type-specific extremities of the NS segment were totally or partially replaced by their counterpart from type C or type A virus, respectively (Figure 1). 

In a type C backbone, no virus was rescued when one or both type A extremities were introduced, and only the wild-type 5’C/3’C virus was recovered (Figure 1). In a type A backbone (Figure 1), no virus harbouring the type C 5’end (5’C/3’C and 5’C/3’A) was rescued. Only viruses with a type A 5’ end (5’A/3’A and 5’A/3’C) could be rescued, but a C-to-U mutation in the 3’C proximal sequence was systematically observed at nucleotide 5 (5’A/3’C(C5U)). Introduction of this reverse substitution into the wild-type 5’A/3’A construct (to generate 5’A/3’A(U5C)) systematically led to a wild-type uracil reversion, confirming the importance of this position. Minigenome experiments previously showed that this position did not modify the transcription/replication processes [10]. Interestingly, this mutation in 5’A/3’C, leading to 5’A/3’C(C5U), restored a proximal panhandle sequence identical to that of the wild-type 5’A/3’A. To demonstrate the necessity of the type specific proximal panhandles to recover viruses, chimeric heterotypic ends were created to include proximal panhandles homotypic of the reverse genetics backbone. Both type A and C viruses with 5’ and 3’ modified NS ends (5’C/3’C-proxA and 5’A/3’A-proxC, respectively) were rescued as efficiently as wild-type viruses (Figure 1). The fact that the proximal panhandle of the unsuccessful constructs is not homotypic strongly suggested that a homotypic proximal panhandle is required.

**Figure 2:**
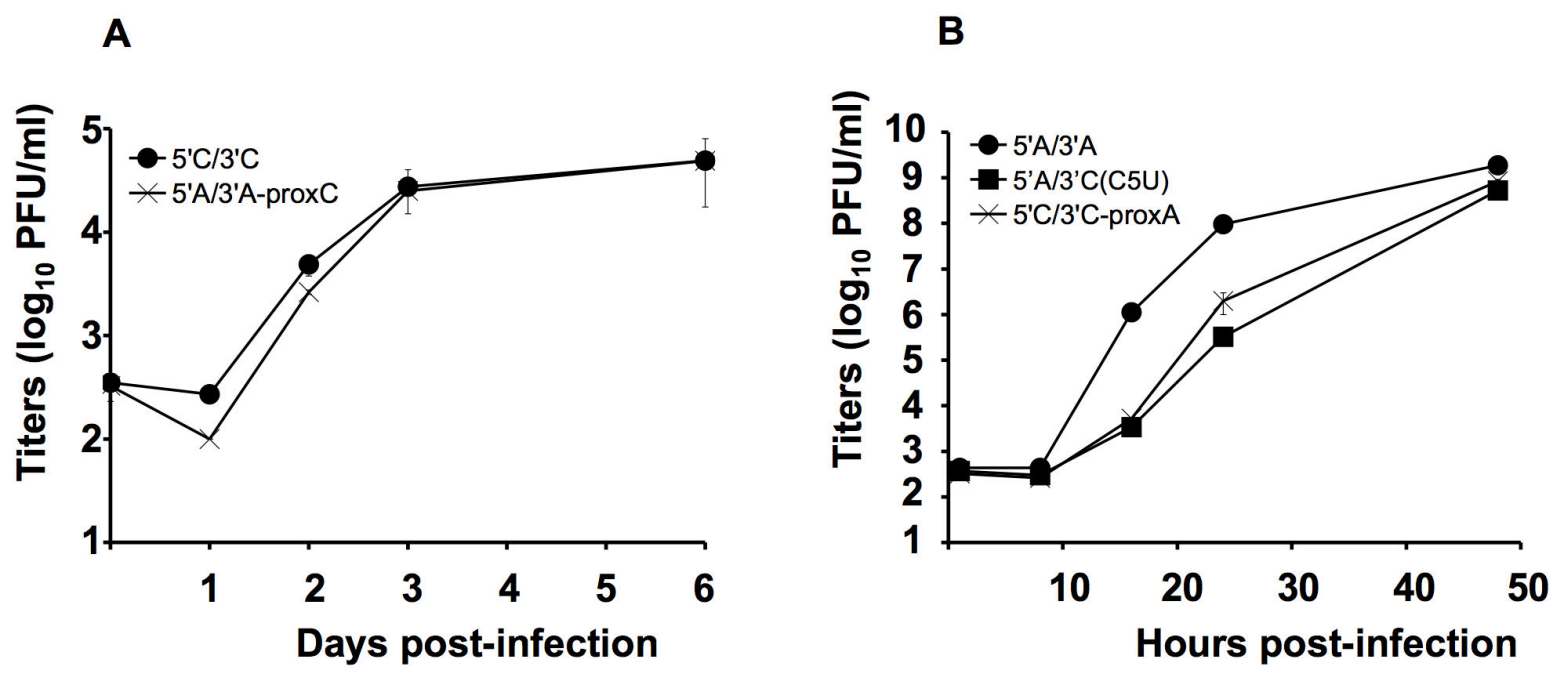
Growth kinetics of rescued influenza viruses. (A) Type C rescued viruses. Infections were performed in SK cells infected at a low m.o.i. (0.001 PFU/cell) in the presence of 0.25 µg/ml of TPCK-trypsin. (B) Type A rescued viruses. Infections were performed in MDCK cells infected at a low m.o.i. (0.001 PFU/cell) in the presence of 1 µg/ml of TPCK-trypsin. Titers were determined by standard plaque assays in duplicate. Results are representative of two independent experiments.

All rescued type C and A viruses were plaque-purified and subsequently amplified in SK and MDCK cells, respectively. Kinetics assays at low multiplicity-of-infection (m.o.i.) were then performed in the respective cell types. No difference was observed for type C viruses; wild-type and 5’A/3’A-proxC grew with similar efficiencies (Figure 2A). The three rescued type A viruses replicated to similar final titers, although both 5’A/3’C(C5U) and 5’C/3’C-proxA were slightly delayed (Figure 2B).

Overall, these results indicated that the major packaging signals were not located within the distal panhandle sequence, since it was possible to rescue the 5’A/3’C(C5U) virus. Thus, the distal sequence of the type C 3’ end was not restrictive for NS segment packaging. This is in agreement with published data showing that NS segment packaging does not require wild-type 3’ end sequences [26].

**Figure 3:**
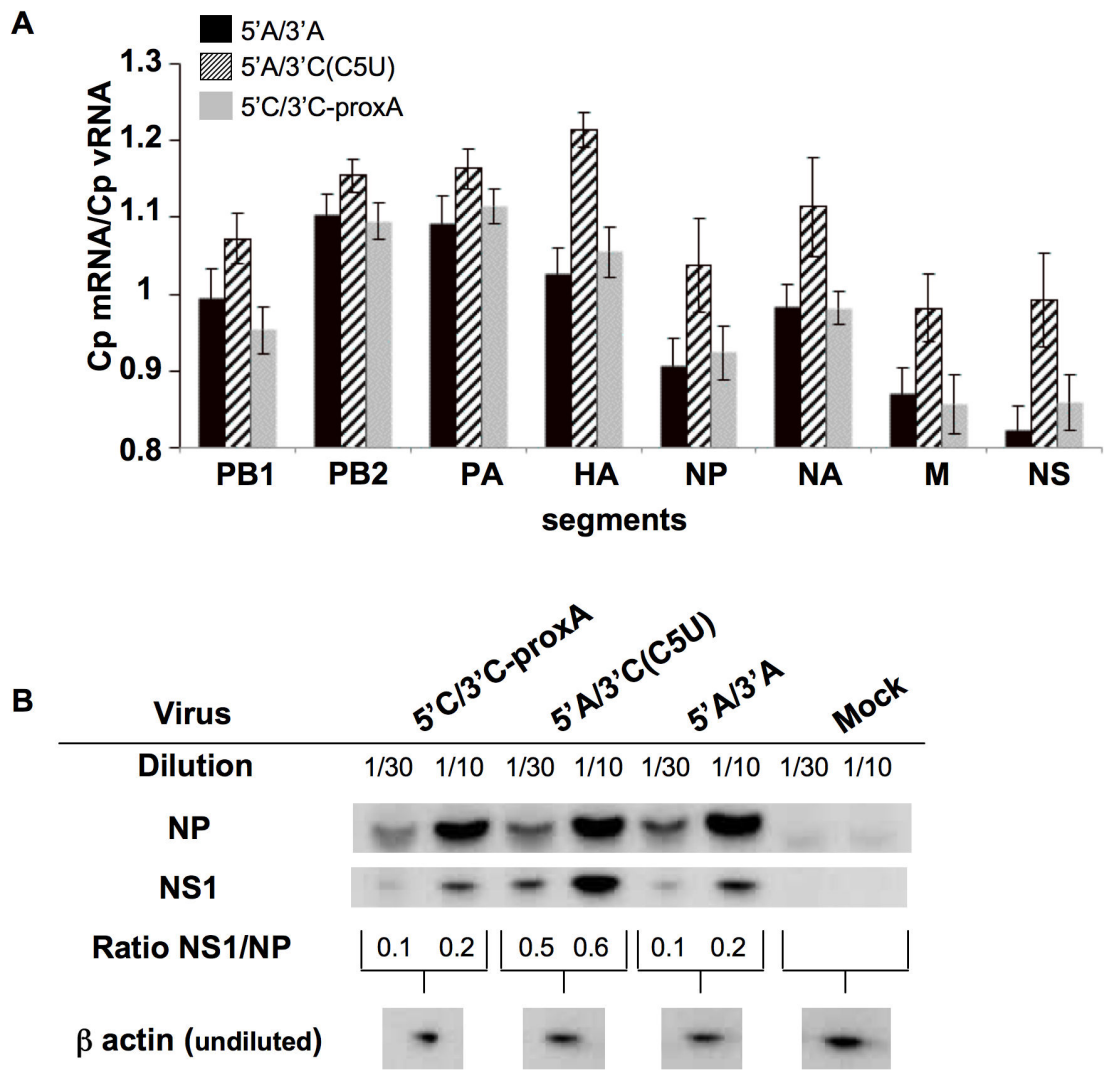
Evaluation of mRNA/vRNA and NS1/NP ratios during single-cycle infection with rescued influenza A viruses. (A) Quantification of vRNA and mRNA in infected cells. Six hours after MDCK infection with rescued influenza A viruses at high m.o.i. (2 PFU/cell), viral vRNA and mRNA levels for each segment were evaluated by specific two-step RT-qPCRs previously described [23]. Results were expressed as the mean of mRNA/vRNA Cp (crossing-point) ratios calculated on data obtained from 12 independent qPCRs (corresponding to two infections, two independent cDNA syntheses per infection and three independent qPCRs for each cDNA). Statistics were performed using the ANOVA test. Black bars: wild-type 5’A/3’A; hatched bars: 5’A/3’C(C5U); grey bars: 5’C/3’C-proxA. (B) Levels of NP and NS1 proteins after infection with the rescued influenza A viruses. Four hours after MDCK infection at a high m.o.i. (2 PFU/cell), cell lysates were analyzed by Western-blot for viral NS1 and NP proteins and for β-actin as cellular control. Following chemiluminescence acquisition with a G. Box (SYNGENE, Cambridge, UK), band densities for viral proteins were determined using GeneTools software (SYNGENE, Cambridge, UK), and were used to calculate NS1/NP ratios for each virus. Due to the high levels of viral protein expression during infection, protein extracts were diluted in uninfected cellular extracts prior to electrophoresis. Results are from one representative assay out of three independent infections.

The putative effect of modifying the extremities on the transcription and replication steps was then studied at an early stage of the viral cycle for the three rescued type A viruses. Levels of mRNA and vRNA were analyzed by RT-qPCR [23] at 6 hours post infection (h.p.i.) at high m.o.i., using RT primers specific for mRNA or vRNA (Figure 3A). Crossing point values were used to evaluate the relative level of both processes, by calculating an mRNA/vRNA ratio. Compared to wild-type 5’A/3’A, significant differences (p<0.05) were found only for 5’A/3’C(C5U), but not for 5’C/3’C-proxA. Interestingly, the CpmRNA/CpvRNA ratio for 5’A/3’C(C5U) was higher than for wild-type 5’A/3’A for all segments, meaning that either more vRNA or less mRNA was synthesized for 5’A/3’C(C5U). The sole difference between 5’A/3’C(C5U) and wild-type 5’A/3’A viruses being the 3’ distal extremity of the NS segment suggested that the level of NS encoded proteins (i.e. NS1 or/and NS2/NEP) was affected at early stages of infection for this virus (5’A/3’C(C5U)). Western-blot was used to evaluate the amount of NS encoded proteins two hours before the observed effect. The amount of NP protein was used to normalize the results. At 4 h.p.i. at high m.o.i., only 5’A/3’C(C5U) showed a clear increase in the production of NS1 protein, as indicated by a higher NS1/NP ratio (Figure 3B). The putative roles of the proteins encoded by the NS segment in viral transcription and replication have been documented: (i) inactivation of NS1 protein reduces the amount of all vRNAs without affecting the amount of mRNAs [27]; (ii) NS1 protein counteracts the effect of a polymerase regulating factor, possibly hnRNP-F [28], that inhibits influenza virus full-length RNA synthesis [29]; and (iii) NS2/NEP protein reduces transcription and increases replication [30]. The latter role of NS2/NEP could be indirect by stimulating the synthesis of virus-derived small viral RNAs, whose depletion was shown to reduce vRNA synthesis [31]. Thus, the decreased mRNA/vRNA ratio observed at 6 h.p.i. at high m.o.i. could reflect an increase in replication, which would be consistent with the higher viral titer for 5’A/3’C(C5U) we observed at that time point (5x10^5^ vs 1.6x10^7^ PFU/ml for wild-type and 5’A/3’C(C5U), respectively).

The most likely hypothesis for the increased amount of 5’A/3’C(C5U) NS proteins is that the level of transcription was affected and that more mRNA was synthesized for 5’A/3’C(C5U) at earlier stages of infection (2-4 h.p.i.). Such a trend, but no significant difference, in NS mRNA quantification after normalization relative to GAPDH was indeed observed between two and four h.p.i. (data not shown). The difference in the base-pairing strength of the distal panhandle formed by the heterotypic extremities in 5’A/3’C(C5U) compared to wild-type, evaluated by scores calculated based on the number and nature of the base-pairs (Figure 1), could favour the attachment of the polymerase. The stability of the duplex formed by the 5’ and 3’ ends was indeed shown to be an important parameter for polymerase binding [32]. A weaker distal panhandle for 5’A/3’C(C5U) (score 7.39 vs 10.74 for wild-type) could facilitate polymerase binding and/or transcription initiation, resulting in slightly more NS mRNA synthesized. This is further supported by the results for the 5’C/3’C-proxA virus, whose distal panhandle base-pairing score is identical to that of wild-type 5’A/3’A (Figure 1), and for which NS1 expression levels (Figure 3B) and mRNA/vRNA ratios (Figure 3A) were similar to those obtained for wild-type. 

The major role of the proximal panhandle in type specificity that we identified and the hypothesized involvement of the distal panhandle in transcription need to be tested on the other segments. It is interesting to note that comparison of the distal panhandles formed by the extremities of each segment for a given virus shows differences in base-pairing scores (data not shown). In particular, regardless of the influenza A virus subtype, the distal panhandle of the HA segment was systematically the weakest one. It would be interesting to determine to what extent the distal panhandle base-pairing strength may have an impact on HA expression levels and consequently influence the HA/NA molecule ratio found at the virion surface [33]. 

## Conclusion

Our approach to understand what in the NS segment non-coding regions was important for type specificity by attempting to rescue type A and C influenza viruses with chimeric NC sequences in the NS segment unraveled the requirement for a type specific proximal panhandle and the role of the distal panhandle in regulation of transcription. Our results thus provide useful information to improve our understanding of influenza A virus biology.
